# Heterozygous β-thalassaemia as a susceptibility factor in mood disorders: excessive prevalence in bipolar patients

**DOI:** 10.1186/1745-0179-1-6

**Published:** 2005-06-01

**Authors:** Alberto Bocchetta

**Affiliations:** 1 Bernard B. Brodie Department of Neurosciences, University of Cagliari, Italy

## Abstract

**Background:**

Previous preliminary reports have suggested potential interactions between microcytic anaemia and mood disorders. In particular, heterozygous β-thalassaemia has been implicated in the bipolar spectrum. This study surveyed relevant haematological parameters in a large sample of psychiatric outpatients with the aim of clarifying previous observations.

**Methods:**

Mean Corpuscular Volume (MCV) was analysed in 1014 consecutive patients diagnosed according to modified Research Diagnostic Criteria (RDC). Haemoglobin electrophoresis and/or chromatography was performed in blood samples from 143 patients with reduced MCV. Prevalence of heterozygous β-thalassaemia was estimated based on the rates of patients with reduced MCV and increased haemoglobin A_2 _concentration.

**Results:**

MCV lower than 80 μ^3 ^was found in greater proportions among bipolar compared with the remaining RDC subgroups (183/732 = 25.0% versus 51/282 = 18.1%; p = 0.02; relative risk = 1.38; Fisher's exact test). This difference can mainly be attributed to heterozygous β-thalassaemia, the estimated prevalence of which was 16.4% among bipolar and 9.9% among non-bipolar subgroups (p = 0.01; relative risk = 1.65).

**Conclusion:**

The results are consistent with the hypothesis that heterozygous β-thalassaemia might play a role as a susceptibility factor in bipolar spectrum disorders in specific populations.

## Background

Our interest in potential interactions between mood disorders of the bipolar spectrum and blood conditions typical of the Mediterranean area, such as glucose-6-phosphate dehydrogenase (G6PD) deficiency and heterozygous β-thalassaemia, was first aroused in the 1980 s. At that time, chromosomes Xq28 and 11p15, where the G6PD gene and the β-globin gene cluster are located, became very popular in psychiatric genetics following the reports of positive linkage with manic-depression [1-3].

We subsequently surveyed G6PD deficiency and heterozygous β-thalassaemia as phenotypic markers to be used in linkage studies [4]. Unexpectedly, we found excessive proportions of the two markers among bipolar patients, especially those with psychotic symptoms [5-7]. Among potential mechanisms underlying the observed associations, we considered linkage disequilibrium between the markers and mood disorder genes located in Xq28 and 11p15. This hypothesis, which at first was most intriguing, weakened considerably when subsequent studies reported diminished evidence of Xq28 or 11p15 linkage [8,9]. Over the following years, the use of phenotypic markers in psychiatric genetics vanished due to the advent of molecular DNA markers. Nevertheless, with regard to heterozygous β-thalassaemia, our interest was periodically revived by the appearance of relevant reports. On the one hand, co-occurrence of the thalassaemic trait and bipolar disorder was reported in several cases from various countries, including Japan, where thalassaemia is very rare [10-13]. On the other hand, a psychiatric population surveyed in Argentina revealed excessive prevalence of microcytic anaemia among patients suffering from recurrent mood disorder [14]. Given the latter series of intriguing reports, an extension of our previous surveys was warranted: this is an updated account of haematological parameters regarding 1014 patients.

## Methods

Outpatients consecutively admitted to the Department of Neuroscience, University of Cagliari, between 1980 and 2000 were studied. The department is one of the reference centres for the management of lithium and related treatments of mood disorders in southern Sardinia. On admission, detailed information was obtained from the patient and from other available sources (such as relatives and referring clinicians) concerning demographic characteristics and lifetime medical and psychiatric history. Moreover, all available records regarding previous prescriptions or hospitalisations were examined. Psychiatric diagnosis was made according to modified Research Diagnostic Criteria (RDC) [15,7]. Routine laboratory tests, including blood count and serum iron concentration were available for 1014 patients. All subjects gave their informed consent to participate in the study.

Additional procedures were used aiming at the identification of heterozygous β-thalassaemia, which is ordinarily diagnosed based on reduced MCV, increased haemoglobin A_2 _(HbA_2_), and normal serum iron concentration. In an initial survey [5], haemoglobin electrophoresis was systematically performed in samples from 180 patients who had attended the centre between April 3rd and May 3rd, 1989. Thereafter, MCV lower than 80 μ^3 ^was chosen as the cut-off for the performing of haemoglobin electrophoresis and/or chromatography (98% of cases with abnormally high HbA_2 _concentration from the initial survey had MCV<80 μ^3^). Overall, electrophoresis and/or chromatography were obtained for 143 patients with MCV<80 μ^3^. Of the latter, 62% had increased HbA_2 _(heterozygous β-thalassaemia); 22% had normal HbA_2 _and normal serum iron (in Sardinia, this pattern has been characterised molecularly as heterozygous α-thalassaemia in the large majority of cases [16]). In the remaining 16%, HbA_2 _concentration was normal but serum iron was reduced, therefore heterozygous β-thalassaemia could not be ruled out (iron-deficiency may maintain HbA_2 _within the normal range).

## Results

Overall, MCV<80 μ^3 ^was found in 23.1% of patients (Table 1). Greater proportions were found among subgroups with bipolar course compared with the remaining subgroups (p = 0.02 at the Fisher's exact test; 183/732 = 25.0% versus 51/282 = 18.1%; relative risk = 1.38). The highest proportion was found in Manic Schizoaffective disorder, predominantly affective subtype (74/219 = 33.8%).

**Table 1:** Reduced Mean Corpuscular Volume (MCV) and Heterozygous β-Thalassaemia in 1014 Psychiatric Outpatients

RDC Diagnosis	Number of Patients	MCV<80 μ^3 ^N (%)	Heterozygous β-Thalassaemia^a^		
			
			Definite	Probable	Total (%)
Manic Schizoaffective	288	88 (30.6)	42	16	58 (20.1)
Bipolar with Mania	269	59 (21.9)	17	21	38 (14.1)
Bipolar with Hypomania	175	36 (20.6)	16	8	24 (13.7)
Depressive Schizoaffective	96	19 (19.8)	6	4	10 (10.4)
Recurrent Major Depression	124	24 (19.4)	7	7	14 (11.3)
Other (Schizophrenia, Minor Depression, etc)	62	8 (12.9)	1	3	4 (6.5)
TOTAL	1014	234 (23.1)	89	59	148 (14.6)

^a^Rates of heterozygous β-thalassaemia are estimated based on the number of definite cases (MCV <80 μ^3 ^and increased HbA_2_) plus the number of probable cases (62 % of patients with MCV <80 μ^3 ^but unknown HbA_2_).

Heterozygous β-thalassaemia (increased HbA_2 _concentration) accounted for the majority of patients with MCV<80 μ^3^. Rates did not vary between the initial survey (27/42 = 64%) and subsequent cases who underwent HbA_2 _analysis (62/101 = 62%). If similar rates were assumed regarding the remaining 91 patients with MCV<80 μ^3 ^but unknown HbA_2 _concentration, prevalence of heterozygous β-thalassaemia in the overall sample could be estimated as at least 16.4% among bipolar and 9.9% among non-bipolar subgroups. A simulation of the Fisher's exact test provided a bipolar/non-bipolar relative risk of 1.65 with a p value of 0.01. Estimated rates of heterozygous β-thalassaemia across psychiatric diagnoses are shown in Table 1. The subgroup of patients with Manic Schizoaffective disorder, predominantly affective subtype (not shown), had the highest estimated rate of heterozygous β-thalassaemia (22%).

The distribution of patients with MCV<80 μ^3 ^and normal HbA_2 _concentration did not vary across psychiatric diagnoses (not shown).

## Discussion

### Reduced Mean Corpuscular Volume and Mood Disorder

In this survey, an impressive 24% of the 952 patients suffering from major mood disorders had an MCV lower than 80 μ^3^. Rates of reduced MCV were lower (13%) in the small subgroup of patients (N = 62) with other psychiatric diagnoses. The direction of results strikingly parallels that from an Argentinean survey [14], which reported a 17.7 % prevalence of microcytic anaemia among 79 patients with recurrent mood disorders compared with 8.0 % prevalence among 25 patients with other psychiatric diagnoses (schizophrenia, anxiety, etc). The lack of similar studies in the literature is surprising given the number of symptoms shared between anaemia and depression (fatigue, asthenia, somatic complaints, etc). On the contrary, many studies have investigated macrocytic anaemia and the effects of vitamin B_12 _deficiency in the CNS.

Prevalence of reduced MCV appeared to follow a hierarchy across the spectrum of mood disorders, according to the presence of psychotic and manic features. Since severity of symptoms may reflect an increased underlying biological/genetic load, the hypothesis of microcytic anaemia as a susceptibility factor is warranted.

### Heterozygous β-Thalassaemia and Mood Disorder

In this survey, variation in prevalence of reduced MCV by psychiatric diagnosis was mostly attributable to heterozygous β-thalassaemia. Data substantially confirmed those from our initial 1989 survey [5], in which haemoglobin electrophoresis was systematically performed in blood samples from 180 patients. The highest rates of heterozygous β-thalassaemia had been found in Manic Schizoaffective Disorder and an apparent bipolar/non-bipolar distinction had already been signalled. In this extended sample, the estimated rates were 16.4 % in bipolar and 9.9 % in non-bipolar subgroups. Results paralleled those from the above mentioned Argentinean survey [14], where heterozygous β-thalassaemia was diagnosed in four of the 51 bipolar patients (7.8 %) and in only one of the 53 remaining cases (1.9 %). Prevalence in the general population in Argentina has been estimated at approximately 5% [14], even if geographical variation is assumed reflecting non-homogeneous ethnic distribution. In Sardinia, 12.6 % is the rate found in the general population [17], but a wide variation has been reported following once malarial morbidity [18].

Potential geographical stratification is a relevant point in the interpretation of our findings: in fact, if mood disorder with psychotic and/or manic features is more prevalent for unknown reasons in once malarial areas in Sardinia, a spurious association with heterozygous β-thalassaemia might have derived. The latter possibility is however challenged by the similarity in results between two independent studies (ours and the Argentinean one). Another approach capable of ruling out a potential geographical bias is the study of segregation between marker and disease within families, given the shared origin of relatives. Indeed, we found a higher than expected concordance between mood disorder and the blood condition within families of our patients with heterozygous β-thalassaemia (to be reported in a different account). A tendency towards familial co-segregation was also inferable by pooling data from published case reports of bipolar disorder and heterozygous β-thalassaemia ascertained in Canada [10], Eire [11], Japan [12], and Australia [13].

In the presence of a true genetic association, different mechanisms may be hypothesised, including linkage disequilibrium between the globin gene and a mood disorder gene closely located in chromosome 11p15 or a direct effect of heterozygous β-thalassaemia in clinical presentation or severity of mood disorder.

Whatever the mechanism, the relevance of heterozygous β-thalassaemia as a susceptibility factor in mood disorder can be inferred from two recently published studies from India [19] and Greece [20]. Since the context was that of studies of psychosocial adjustment of parents of (or at risk of bearing) children with Cooley's anaemia, attention was mostly directed to current depression and anxiety (rather than to history of bipolar or schizoaffective disorder, if any). Moreover, the potential role of the mild or silent blood condition in parents was not taken into account. In the Indian study [19], depression was diagnosed in 70% of parents (19 mothers and 11 fathers) accompanying their thalassaemic child for blood transfusion. Unfortunately no group of parents of children suffering from other severe chronic diseases was included for comparison.

The Greek study [20] reported clinically relevant depression in 10.1% of 159 women with heterozygous β-thalassaemia undergoing chorionic villus sampling due to increased possibility of carrying an embryo with Cooley's anaemia (spouses were β-thalassaemic carriers too). As comparison groups, 150 women undergoing karyotyping for risk of trisomy and 309 undergoing a routine first trimester scan were studied. Depression was found in 4.7 % and 0.3 %, respectively.

In another Greek study [21], more psychiatric disorders, including a broad range of DSM-III-R diagnoses, were found among 71 siblings of subjects with Cooley's anaemia compared with 71 control subjects. In the age group from 10 to 19 years, the difference was significant (61% compared with 37%). Even if the latter data are intriguing, no conclusion can be drawn regarding the role of heterozygous β-thalassaemia in this sample, because haematological data of participants were not envisaged. However, since the expected proportion of thalassaemic carriers among siblings of subjects with Cooley's anaemia is 2/3, similar samples would be very informative. Inclusion of older age groups, when mood disorders are better characterised, would be preferable.

## Conclusion

The results from this survey and the series of intriguing literature reports are consistent with the hypothesis that heterozygous β-thalassaemia might play a role as a susceptibility factor in mood disorders, particularly of the bipolar spectrum. Since the evidence cannot be considered established, further studies are warranted. Potential approaches include: a) replication surveys of microcytic anaemia and the thalassaemic trait in psychiatric populations from high prevalence areas; b) surveys of bipolar spectrum disorders among subjects with an already established diagnosis of heterozygous β-thalassaemia (such as relatives of patients with Cooley's anaemia or participants in specific screening programs); c) family studies of patients with bipolar disorder and an established thalassaemic trait.
